# An innovative, sustainable, and environmentally friendly approach for wheat drought tolerance using vermicompost and effective microorganisms: upregulating the antioxidant defense machinery, glyoxalase system, and osmotic regulatory substances

**DOI:** 10.1186/s12870-024-05550-2

**Published:** 2024-09-17

**Authors:** Neveen B. Talaat, Sameh A. M. Abdel-Salam

**Affiliations:** 1 Department of Plant Physiology, Faculty of Agriculture, Cairo University, Giza, Egypt; 2 Department of Animal Production, Faculty of Agriculture, Cairo University, Giza, Egypt

**Keywords:** Antioxidant capacity, Effective microorganisms, Methylglyoxal detoxification machinery, Organic solutes, Vermicompost, Water deficit stress, *Triticum aestivum* L

## Abstract

**Background:**

Vermicompost contains humic acids, nutrients, earthworm excretions, beneficial microbes, growth hormones, and enzymes, which help plants to tolerate a variety of abiotic stresses. Effective microorganisms (EM) include a wide range of microorganisms’ e.g. photosynthetic bacteria, lactic acid bacteria, yeasts, actinomycetes, and fermenting fungi that can stimulate plant growth and improve soil fertility. To our knowledge, no study has yet investigated the possible role of vermicompost and EM dual application in enhancing plant tolerance to water scarcity.

**Methods:**

Consequently, the current study investigated the effectiveness of vermicompost and EM in mitigating drought-induced changes in wheat. The experiment followed a completely randomized design with twelve treatments. The treatments included control, as well as individual and combined applications of vermicompost and EM at three different irrigation levels (100%, 70%, and 30% of field capacity).

**Results:**

The findings demonstrated that the application of vermicompost and/or EM significantly improved wheat growth and productivity, as well as alleviated drought-induced oxidative damage with decreased the generation of superoxide anion radical and hydrogen peroxide. This was achieved by upregulating the activities of several antioxidant enzymes, including superoxide dismutase, catalase, peroxidase, ascorbate peroxidase, glutathione peroxidase, monodehydroascorbate reductase, dehydroascorbate reductase, and glutathione reductase. Vermicompost and/or EM treatments also enhanced the antioxidant defense system by increasing the content of antioxidant molecules such as ascorbate, glutathione, phenolic compounds, and flavonoids. Additionally, the overproduction of methylglyoxal in water-stressed treated plants was controlled by the enhanced activity of the glyoxalase system enzymes; glyoxalase I and glyoxalase II. The treated plants maintained higher water content related to the higher content of osmotic regulatory substances like soluble sugars, free amino acids, glycinebetaine, and proline.

**Conclusions:**

Collectively, we offer the first report that identifies the underlying mechanism by which the dual application of vermicompost and EM confers drought tolerance in wheat by improving osmolyte accumulation and modulating antioxidant defense and glyoxalase systems.

**Supplementary Information:**

The online version contains supplementary material available at 10.1186/s12870-024-05550-2.

## Introduction

Wheat (*Triticum aestivum* L.) is the world’s most popular staple food crop [1, 2]. However, water stress reduces its annual yield by 20–30% [3]. Water scarcity is the most common abiotic stress that threatens agricultural sustainability [4, 5]. By 2050, it is expected that water scarcity would affect more than half of the world’s regions [6]. It can alter carbon and nitrogen metabolic processes, disrupt water relations, and impair photosynthetic activity, resulting in lower crop yield [7, 8]. Furthermore, it causes oxidative damage by increasing the generation of reactive oxygen species (ROS), which harms biomolecules and cellular membrane [9–11]. Plants used their antioxidant defense system, which is made up of both enzymatic and non-enzymatic components, to combat the overproduction of harmful ROS [12, 13]. Nonetheless, the quantity of excess ROS that the plant’s antioxidant defense system detoxifies determines the plant’s capacity to endure a drought [5, 10]. Another significant cytotoxic substance that is produced in greater quantities during drought is methylglyoxal (MG) [14]. The MG detoxifying mechanism depends on two essential enzymes; glyoxalase I and glyoxalase II [4]. Additionally, in response to drought stress, plants generate a variety of osmoprotectants, including soluble sugars, free amino acids, glycinebetaine, and proline, which maintain cell turgor pressure, assist osmotic adjustment, preserve cellular integrity, and regulate redox potential [15, 16].

In dry and semiarid areas, the supply of water and nutrients in accordance with the sustainable agriculture principles is more challenging [17]. Vermicompost is an environmentally friendly organic source for sustaining soil fertility since it contains micro- and macro-nutrients, amino acids, humic acid, vitamins, plant growth regulators, and beneficial microbes [18]. Previous studies have shown that applying vermicompost dramatically boost plant growth and production under water stress circumstances by enhancing soil water capacity and managing water absorption [19–21]. Vermicompost was shown to enhance the plant’s morpho-physiological characteristics and antioxidant capability in drought-stressed environments [22–26]. In line with its role under water deficit conditions, studies by [21, 27] show that it improves soil physicochemical properties and protects plants by providing appropriate moisture content. Thus, enriching agronomic soil with vermicompost could be an efficient and environmentally safe technology to improve soil quality and wheat crop production in water-scarce areas.

Effective microorganisms (EM) have recently received a lot of interest in sustainable agriculture due to their capacity to boost plant growth and productivity under abiotic stress [28, 29]. EM is made up of several various types of useful microorganisms, including photosynthetic bacteria, lactic acid bacteria, yeast, actinomycetes, and fermenting fungi [30]. EM has been shown in earlier research to positively impact plant development and yield in water-scarce environments [31, 32]. Actually, under stressful circumstances, EM treatment can boost the buildup of antioxidant molecules and osmolytes, improve the uptake of nutrients, preserve the stability of the cell membrane, and improve the water use efficiency [28, 30, 33–35]. However, limited information is known about how EM contributes to wheat plants’ ability to withstand drought.

Enhancing wheat productivity in areas with limited water supply is crucial for global food security. Using several beneficial amendments such as vermicompost or EM has shown positive results in alleviating the negative impacts of drought stress [21, 25, 27, 31, 32]. To the best of our knowledge, this is the first study looking into the potential benefits of applying vermicompost and EM dual application to help wheat recover from drought-induced changes. The current study intends to evaluate the possible use of vermicompost and/or EM treatments in improving wheat drought tolerance, including the stimulation of antioxidant enzyme activity, antioxidant molecule content, and glyoxalase system enzyme activity, as well as the buildup of osmotic substances. Based on our findings, we hypothesize that the combination of vermicompost and EM protects wheat plants from drought via modifying antioxidant defense and glyoxalase systems, as well as boosting osmolyte accumulation. Here, we devised and implemented an innovative, sustainable, and environmentally friendly technique to reduce the effects of water scarcity on wheat productivity.

## Materials and methods

### Plant material

Wheat seeds (*Triticum aestivum* L. cv. Sakha 95) were obtained from Egypt’s Wheat Research Department, Agriculture Research Centre, Ministry of Agriculture and were used as a plant material to study the influence of vermicompost and/or EM applications on wheat plants grown under water deficiency.

### Pot experiment

Pot experiment was carried under natural conditions with 60 ± 5% relative humidity and an average temperature of 22/16 ± 2 °C (day/night) in a greenhouse at Cairo University’s Faculty of Agriculture’s Plant Physiology Department. The 30 cm diameter and 35 cm height pots were filled with 15 kg of clay loamy soil (37% sand, 28% silt, and 35% clay). Ammonium nitrate (33.5% N), calcium superphosphate (15.5% P_2_O_5_), and potassium sulfate (48% K_2_O) were applied at rates of 2.0, 2.0, and 0.5 g pot^− 1^, respectively. Additionally, 30 days after planting, 2.0 g pot^− 1^ of ammonium nitrate was added.

### Drought stress treatments

Pots were divided into three groups before sowing. The first group served as a control [well-watered (WW; 100% of field capacity)], and the other two as two levels of irrigation treatments [water deficit stress (WD1; 70% of field capacity) and water deficit stress (WD2; 30% of field capacity)]. Soil water content (SWC) was 15.5%, 10.8%, and 4.6%, respectively, calculated as SWC % = [(FW-DW)/DW] × 100 [36]. All irrigation treatments were implemented from grain sowing to grain filling.

### Experimental design

Twelve treatments were used, including three levels of irrigation (100, 70, and 30% of field capacity) and four treatments [control, as well as individual and combined applications of vermicompost and EM]. The experiment was set up in a completely randomized design, with two factors (water deficit stress and application treatments) and four replicates.

### Preparation of vermicompost

For vermicompost production, a 5 m^2^ area was considered; then, well-rotted animal manure (rabbit + horse manure) was spread over a 50 m long, 1 m wide, and 40 cm thick area. To lessen the salt, well-rotted animal manure was washed away with water prior to production. After 1 day when the bed moisture reached 65–70%, 8 to 10 kg of epigeic earthworm species; *Eisenia andrei*, *Eisenia fetida*, *Eudrilus eugeniae*, *Lumbricus rubellus*, and *Perionyx excavatus* were added per ton of animal manure. Daily watering was planned to maintain an appropriate moisture level (60–70%). In addition, an effort was made to keep the ambient temperature at 23 ± 3 °C. The vermicompost was stirred and aerated with a rake every seven to ten days to make sure there was enough air and oxygen inside the vermicompost, which was ready after three months. Prior to harvesting, irrigation was turned off. Due to the moisture in the lower layers, the earthworms moved to the lower portion when the top layer dried. Vermicompost was consequently taken out of the top layers. The soil was thoroughly mixed with the vermicompost at a ratio of (1:4; vermicompost: soil) before planting.

### Soil and vermicompost analysis

Using a Mettler pH FE28-Standard, the pH of soil and vermicompost was detected at a 1:2.5 ratio (soil/water, m/v). The titration with potassium dichromate was used to measure the organic matter. Total nitrogen was determined using the Kieldahl apparatus, total phosphorus was measured using the sodium hydroxide alkali fusion–molybdenum antimony colorimetric resistance method, total potassium was detected using a flame photometer (Sherwood Flame photometer, Model-410; Sherwood Scientifics, Ltd., Cambridge UK), available nitrogen was determined using the alkaline hydrolysis diffusion method, available phosphorus was measured using the NaHCO_3_ extraction-molybdenum antimony colorimetric resistance method, and rapidly potassium was detected using the NH_4_OAc extraction-flame photometry method [37]. Iron, zinc, and copper element analyses were performed using an atomic-absorption spectrophotometer (Unicam 989-AA Spectrometer-UK). The physio-chemical properties of the soil and vermicompost used in the current experiment are shown in Table 1.

**Table 1:** Physio-chemical properties of the soil and vermicompost used in the experiment

Characteristic	Soil	Vermicompost
Moisture content (%)	2.41 ± 0.09	4.33 ± 0.07
pH	7.20 ± 0.15	7.79 ± 0.11
Organic carbon (%)	0.83 ± 0.01	38.37 ± 0.97
Organic matter (%)	2.11 ± 0.06	61.98 ± 1.01
Total nitrogen (%)	0.22 ± 0.03	3.37 ± 0.10
Total phosphorus (%)	0.06 ± 0.00	2.61 ± 0.09
Total potassium (%)	0.41 ± 0.04	3.07 ± 0.11
Available nitrogen (mg Kg^− 1^)	33.14 ± 0.66	564.98 ± 10.03
Available phosphorus (mg Kg^− 1^)	28.27 ± 1.03	224.49 ± 6.45
Available potassium (mg Kg^− 1^)	46.41 ± 1.29	471.48 ± 10.66
Iron (ppm)	5.90 ± 0.09	1399 ± 16.33
Zinc (ppm)	2.00 ± 0.07	150 ± 3.23
Copper (ppm)	1.10 ± 0.04	41 ± 1.14

Values are means ± standard error (*n* = 4)

### Effective microorganisms’ application

Ten days after sowing, the EM treatment was applied to plants from each irrigation level. It was used in the EM1 formulation developed by Egypt’s Ministry of Agriculture and Land Reclamation. EM1 contained 3.3 × 10^4^ Colony Forming Unit (CFU) mL^− 1^ of photosynthetic bacteria (*Rhodopseudomonas palustrus* and *Rhodobacter spaeroides*), 1.3 × 10^7^ CFU mL^− 1^ of lactic acid bacteria (*Lactobacillus plantarum*,* Lactobacillus casei*, and *Streptoccus lactis*), 1.3 × 10^4^ CFU mL^− 1^ of yeast (*Saccharomyces cerevisiae* and *Candida utilis*), 10^5^ CFU mL^− 1^ actinomycetes (*Streptomyces albus* and *Streptomyces griseus*), and 10^5^ CFU mL^− 1^ fermenting fungi (*Aspergillus oryzae*,* Penicillium sp.*, and *Mucor hiemalis*)]. A 1:1000 (EM: water) dilution of the EM1 stock solution was performed. During irrigation, 7 mL pot^− 1^ from this diluted solution was sprayed on the plant and soil surface as recommended by Egypt’s Ministry of Agriculture and Land Reclamation.

### Growth and yield analysis

In 70-day-old wheat plants, plant height, leaves number, total leaf area, and shoot dry weight were measured. The total leaf area was measured using a portable leaf area meter (LI-COR 3000, Lambda Instruments Corporation, Lincoln, Nebraska, USA). After being dried in an oven for 48 h at 70 °C, the shoot dry weight was calculated. Four replicates, each with six plants taken from the same pot, were used to collect the data. At maturity, plants in each treatment were harvested to measure the number of grains and grain yield.

In wheat plants that were 70 days old, all the following physiological and biochemical characteristics were identified. Four replicates, each with six plants taken from the same pot, were used to collect the data.

### Determination of superoxide radical (O_2_^• —^), hydrogen peroxide (H_2_O_2_), and lipid peroxidation

The O_2_^• —^ levels were estimated according to [38] with some modifications. One milliliter of hydroxylammonium chloride (1 mM) was added into 0.5 mL of the supernatant and incubated for 1 h at 25 °C. 1 mL 4-aminobenzenesulfonic acid (17 mM) and 1 ml α-naphthylamine (7 mM) were added and the specific absorption was determined at 530 nm. Following the directions on the H_2_O_2_ and malondialdehyde (MDA) kits, 0.1 g of fresh wheat leaves were ground in a mortar with 900 µL of buffer to estimate H_2_O_2_ and MDA, using the procedure described by [39]. At 405 nm and 532 nm, respectively, the contents of H_2_O_2_ and MDA were measured.

### Extraction and estimation of antioxidant enzyme activities

The protein concentration of each sample was measured following the method of [40] using bovine serum albumin as standard. To extract enzymes, 0.5 g of wheat leaf tissue was homogenized in 1 mL of 50 mM ice-cold K-P buffer (pH 7.0) containing 100 mM KCl, 1 mM AsA, 5 mM β-mercaptoethanol, and 10% (w/v) glycerol. The homogenates were centrifuged at 11,500 × g for 15 min, and the supernatants were used to determine the enzyme activity. Superoxide dismutase (SOD; EC: 1.15.1.1) activity was assayed as described previously by [41]. One unit of SOD was defined as the amount of enzyme required to cause 50% inhibition in the rate of NBT photo reduction. The absorbance was measured at 560 nm. Catalase (CAT; EC: 1.11.1.6) activity was assayed following the method of [42] by monitoring the decrease in absorbance at 240 nm for 1 min caused by the decomposition of H_2_O_2_. Peroxidase (POD; EC: 1.11.1.7) was assayed by the method previously described by [43]. The initial and final absorbance was recorded at 470 nm for 2 min. Ascorbate peroxidase (APX; EC: 1.11.1.11) activity was assayed following the method of [44]. The reaction buffer solution contained K-P buffer (pH 7.0), AsA, H_2_O_2_, and EDTA. The decrease in absorbance was observed at 290 nm for 1 min using an extinction coefficient of 2.8 mM^–1^ cm^–1^. Monodehydroascorbate reductase (MDHAR; EC: 1.6.5.4) activity was determined by the method of [45]. The activity was calculated from the change in absorbance at 340 nm for 1 min using an extinction coefficient of 6.2 mM^–1^ cm^–1^. Dehydroascorbate reductase (DHAR; EC: 1.8.5.1) activity was determined by the procedure of [44]. The activity was calculated from the change in absorbance at 265 nm for 1 min using an extinction coefficient of 14 mM^–1^ cm^–1^. Glutathione reductase (GR; EC: 1.6.4.2) activity was measured following the method of [42]. The reaction was initiated with GSSG, and the decrease in absorbance at 340 nm was recorded for 1 min. The activity was calculated using an extinction coefficient of 6.2 mM^–1^ cm^–1^. Glutathione peroxidase (GPX; EC: 1.11.1.9) activity was assayed using the method of [46]. The oxidation of NADPH was recorded at 340 nm for 1 min, and the activity was calculated using extinction coefficient 6.62 mM^–1^ cm^–1^.

### Ascorbate and dehydroascorbate assay

Total and reduced ascorbate (AsA) content were determined according to the method of [47] with modifications. Total and reduced AsA content were assayed spectrophotometrically at 265 nm in 100 mM K-P buffer with 1.0 U of ascorbate oxidase. To calculate AsA, a specific standard curve of AsA was used. The oxidized form of ascorbate (DHA, dehydroascorbate) was measured using the formula DHA = Total AsA − Reduced AsA.

### Glutathione and oxidized glutathione assay

Glutathione (GSH) was assayed according to [48], utilizing aliquots of supernatant neutralized with 0.5 M K-P buffer. Based on enzymatic recycling, glutathione is oxidized by DTNB and reduced by NADPH in the presence of GR, and glutathione content is evaluated by the rate of absorption changes at 412 nm. Oxidized glutathione (GSSG) was determined after removal of GSH by 2-vinylpyridine derivatization. Standard curves with known concentrations of GSH and GSSG were used for the quantification.

### Phenolic and flavonoids compounds determination

Total phenolic compounds and total flavonoids were measured as described by [49]. Briefly, 0.2 g fresh wheat leaves were extracted with a mixture of cold 70% (v/v) methanol, 2% (v/v) formic acid, and 28% (v/v) ethanol. The homogenate was centrifuged at 10,000 g for 10 min at 4 °C. Total phenolic compounds were assayed quantitatively by A_765_ with Folin-Ciocalteau reagent. The absorption of total flavonoids was measured at 510 nm. The concentrations of phenols and flavonoids were represented as mg caffeic acid g^− 1^ DW and mg rutin g^− 1^ DW, respectively.

### Methylglyoxal content and glyoxalase activity assay

Methylglyoxal (MG) was measured following the method of [50]. The MG content was calculated using a standard curve of known concentration. Glyoxalase I (Gly I; EC: 4.4.1.5) assay was carried out according to [42]. The activity was calculated using the extinction coefficient of 3.37 mM^–1^ cm^–1^. Glyoxalase II (Gly II; EC: 3.1.2.6) activity was determined according to the method of [51]. The activity was calculated using the extinction coefficient of 13.6 mM^–1^ cm^–1^.

### Determination of osmolytes

Total free amino acids were determined with the ninhydrin reagent method [52]. The absorbance was evaluated at 570 nm. Total soluble sugars were measured by utilizing Anthrone Colorimetry’s established protocol [53]. The final concentration of soluble sugar was calculated from the absorbance values read at 630 nm wavelength using a microplate (Infinite-200 Tecan, Switzerland) and compared with a standard curve. Proline content was measured according to [54]. The absorbance of the aspired toluene fraction was measured at 520 nm. Glycinebetaine was determined by the method of [55] and the absorption value was determined at 365 nm.

### Estimation of leaf water content

Leaf relative water content (RWC) was quantified in accordance with the established protocol [16]. The fresh weight (FW) was measured immediately after harvest, while dry weight (DW) was measured after drying the leaf for 72 h at 60 ºC.

### Statistical analysis

The obtained data was analyzed using the two-way variance analysis (ANOVA). A completely randomized design with four replications was used to analyze the data. Because the results of the two seasons followed a similar pattern, a combined analysis was performed on them. To determine the statistical significance of the means at *p <* 0.05, the least significant difference (LSD) test was used. For data analysis, the SAS software (SAS Inc., Cary, NC) was used. The data are presented as means ± standard error (SE).

## Results

### Wheat growth and productivity

**Fig. 1:**
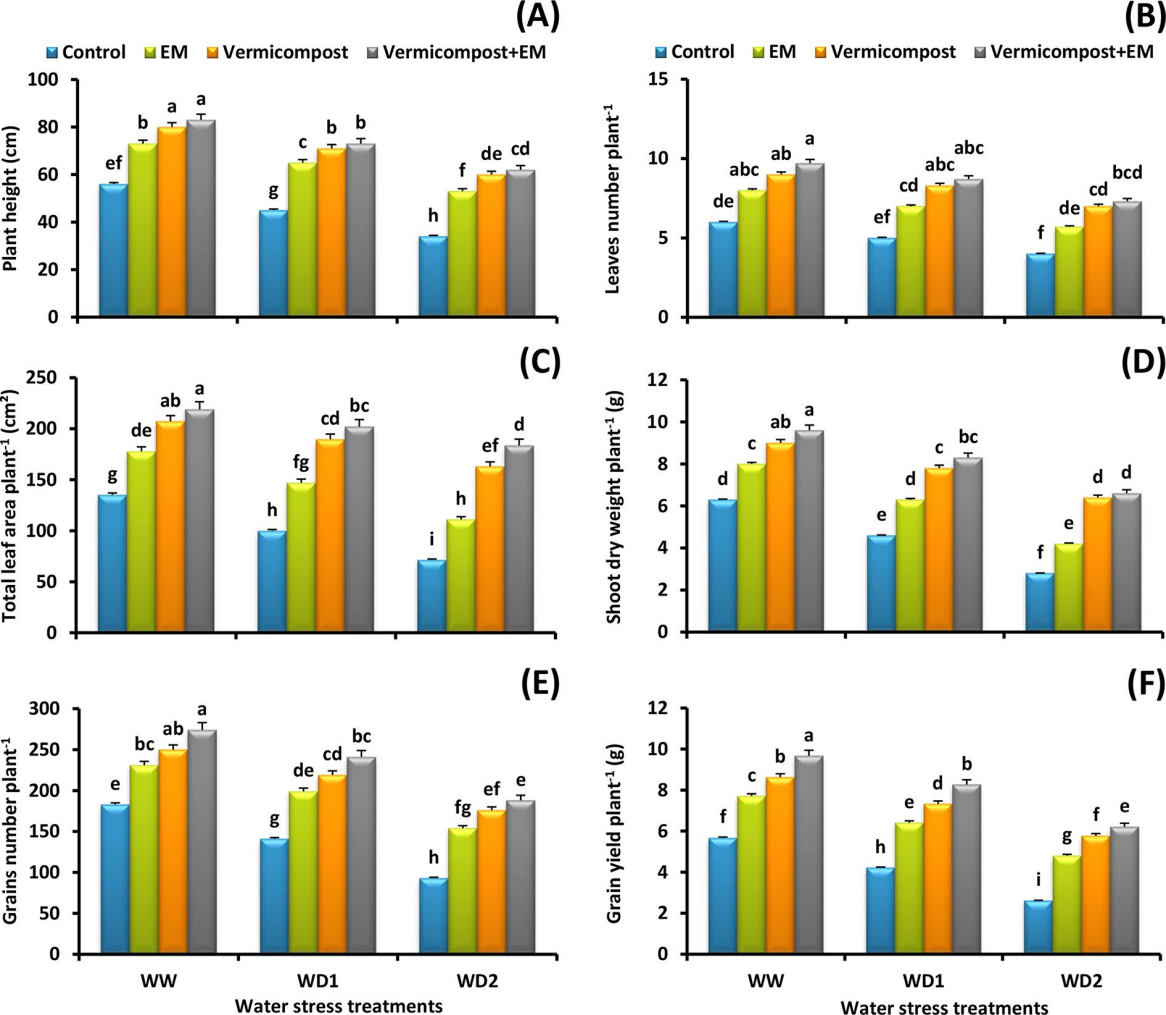
Effect of effective microorganisms (EM), vermicompost, and their interaction on the: **A** plant height (cm), **B** leaves number plant^− 1^, **C** total leaf area plant^− 1^ (cm^2^), **D** shoot dry weight plant^− 1^ (g), **E** grains number plant^− 1^ (g), and **F** grain yield plant^− 1^ (g) of wheat plants grown under different water stress conditions [well-watered (WW), water deficit stress (WD1; 70% of field capacity), and water deficit stress (WD2; 30% of field capacity)]. Vertical bars represent ± standard error of the mean (*n* = 4). Different letters indicate significant differences between treatments at *p* < 0.05 level according to LSD test

The obtained results imply that drought stress significantly (*p* < 0.05) hampered wheat growth and productivity compared to well-watered condition (Fig. 1A-F, Table S). A significant fall in shoot height (39%), leaves number (33%), total leaf area (47%), shoot dry weight (56%), grains number (49%), and grain yield (54%) values was evident in plants grown under a severe (30% FC) water deficit condition relative to the non-stressed plants. Contrarily, applying vermicompost and/or EM alleviated the drought-induced reduction in these parameters, and the dual application appeared to be much more effective for alleviating deleterious effects of water-scarce conditions, reflecting the important role of vermicompost and EM addition.

### ROS (H_2_O_2_ and O_2_^• —^) generation

**Fig. 2:**
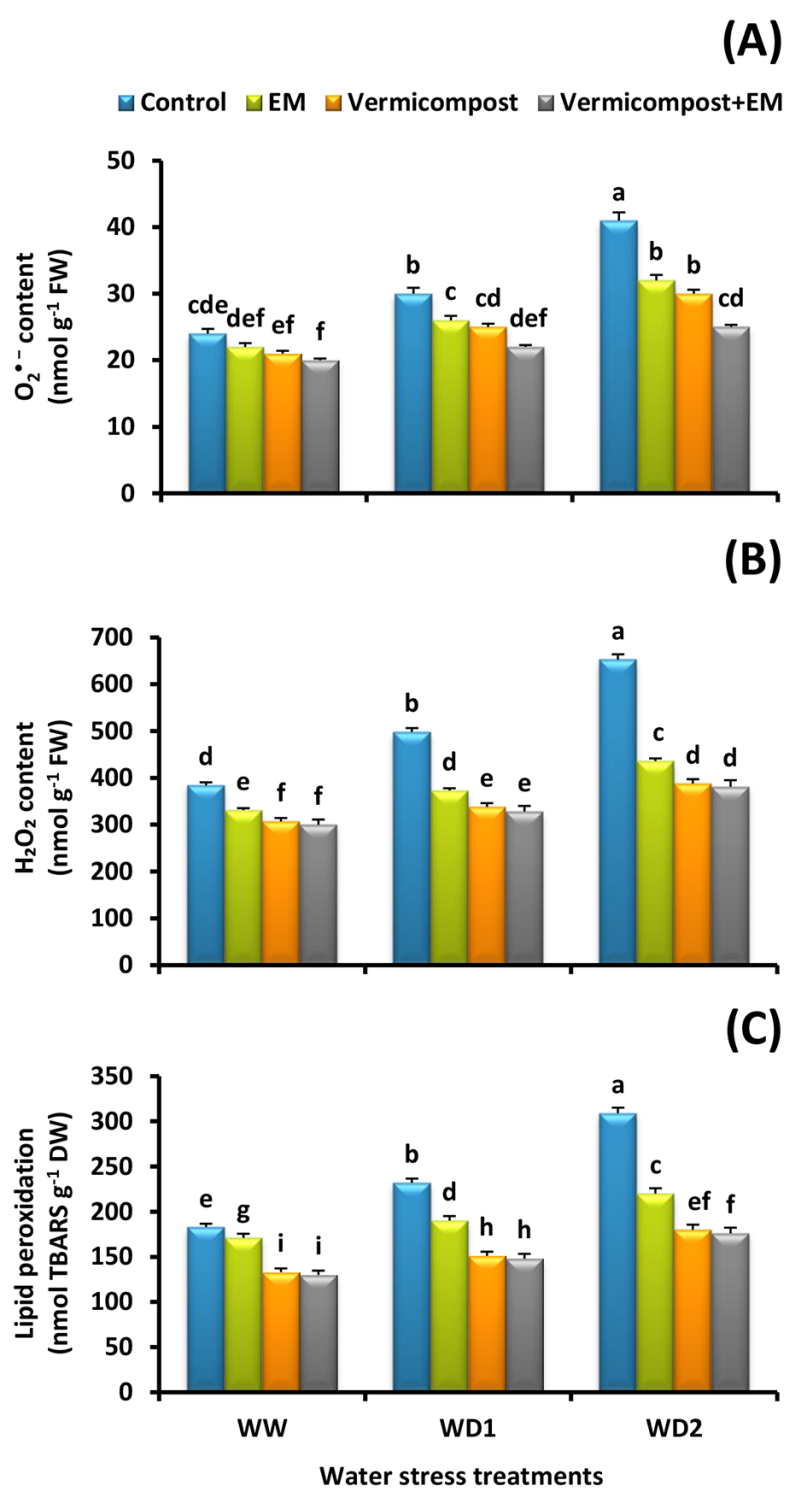
Effect of effective microorganisms (EM), vermicompost, and their interaction on the: **A** superoxide (O_2_^• −^) content, **B** hydrogen peroxide (H_2_O_2_) content, and **C** lipid peroxidation in leaves of wheat plants grown under different water stress conditions [well-watered (WW), water deficit stress (WD1; 70% of field capacity), and water deficit stress (WD2; 30% of field capacity)]. Vertical bars represent ± standard error of the mean (*n* = 4). Different letters indicate significant differences between treatments at *p* < 0.05 level according to LSD test

To investigate if vermicompost and/or EM treatments alleviate drought-induced oxidative stress, the generation of H_2_O_2_ and O_2_^• −^ in wheat leaves was detected. The results revealed that as the stress intensity increased, the H_2_O_2_ and O_2_^• −^ bulidup increased. A severe (30% FC) water stress condition significantly increased H_2_O_2_ and O_2_^• −^ content by 70 and 71%, respectively compared to control, whereas vermicompost and/or EM treatments significantly decreased their content (Fig. 2A and B). The best response was registered with vermicompost and EM combined treatment. It significantly (*p* < 0.05) alleviated the detrimental effects generated under water deficiency and decreased the content of H_2_O_2_ by 22, 34, and 42%; and O_2_^• −^ by 17, 27, and 39%; compared to the untreated plants under well-watered and water-stressed (70% and 30% FC) conditions, respectively.

### Lipid peroxidation

In line with ROS, plant oxidative stress marker (MDA content) was also raised as the water deficit stress increased, reaching maximum levels in plants grown under a severe (30% FC) water deficit condition. However, MDA accumulation in stressed plants treated with vermicompost and/or EM was lower than that in stressed untreated plants (Fig. 2C). The maximum ameliorative effect was detected by vermicompost and EM co-application. In comparison to the untreated plants, combined treatment under the severe (30% FC) water stress condition significantly (*p* < 0.05) decreased the content of MDA (43%) in wheat leaves.

### Antioxidant enzyme activity

**Fig. 3:**
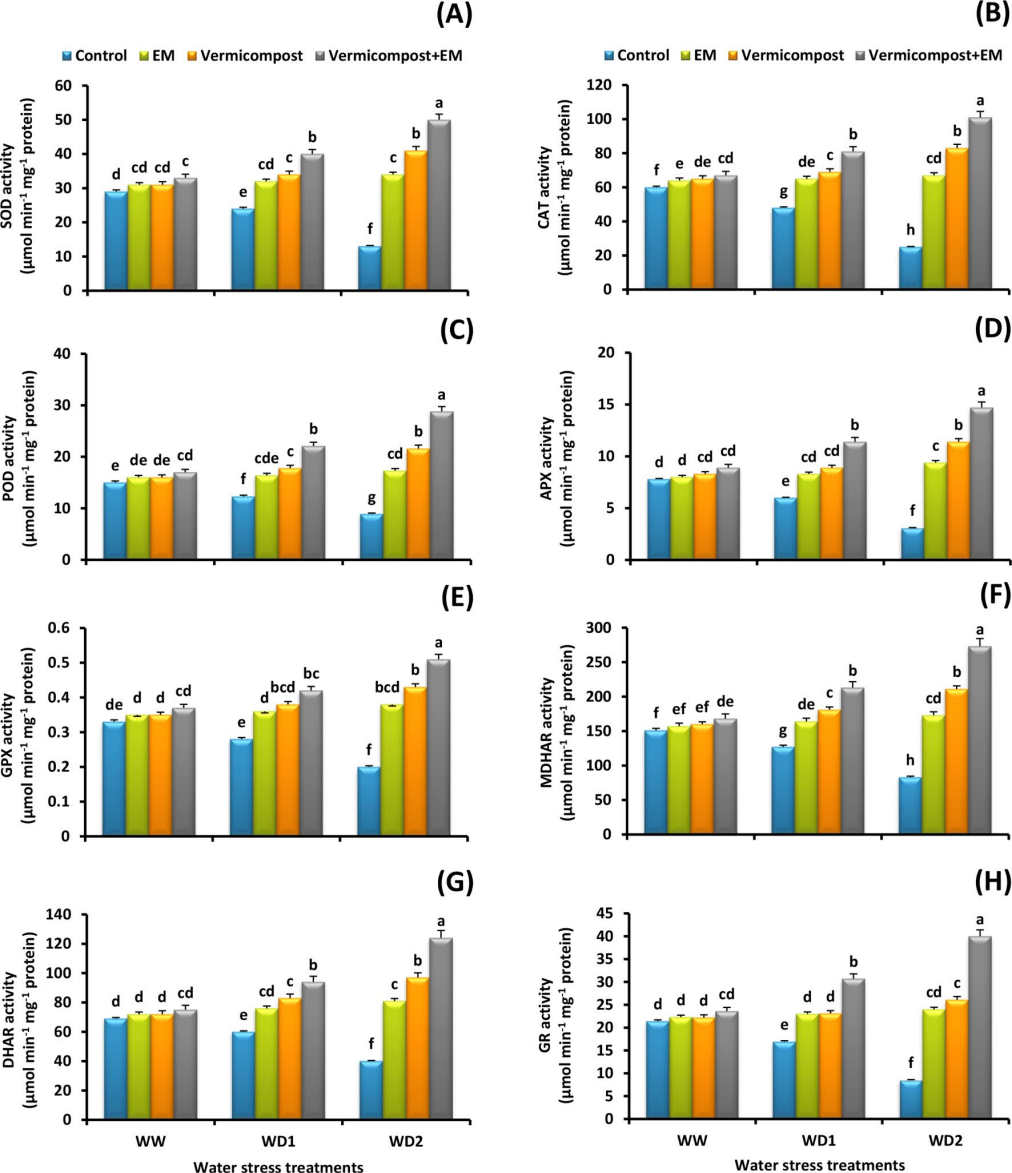
Effect of effective microorganisms (EM), vermicompost, and their interaction on the: **A** superoxide dismutase (SOD), **B** catalase (CAT), **C** peroxidase (POD), **D** ascorbate peroxidase (APX), **E** glutathione peroxidase (GPX), **F** monodehydroascorbate reductase (MDHAR), **G** dehydroascorbate reductase (DHAR), and **H** glutathione reductase (GR), activities in leaves of wheat plants grown under different water stress conditions [well-watered (WW), water deficit stress (WD1; 70% of field capacity), and water deficit stress (WD2; 30% of field capacity)]. Vertical bars represent ± standard error of the mean (*n* = 4). Different letters indicate significant differences between treatments at *p* < 0.05 level according to LSD test

To explicate how vermicompost and/or EM treatments eliminate the adverse effects of water stress, the activity of several antioxidant enzymes were quantified. Our results show that the plants exposure to the water deficit conditions had lower antioxidant enzyme activity than the control ones; the decrease was more severe at 30% FC water deficit condition that resulted in 55, 58, 41, 60, 45, 42, 60, and 39% reduced in the activity of SOD, CAT, POD, APX, MDHAR, DHAR, GR, and GPX, respectively relative to the non-stressed plants. Importantly, under drought stress, vermicompost and/or EM applications significantly (*p* < 0.05) upregulated their activities (Fig. 3A-H). The maximum activities were detected by vermicompost and EM co-application. In comparison to the untreated plants, an 285, 304, 224, 374, 229, 210, 371, and 155% increase in SOD, CAT, POD, APX, MDHAR, DHAR, GR, and GPX activity was detected with the combined treatment under the severe (30% FC) water stress condition in wheat leaves, respectively.

### Non-enzymatic antioxidant content

**Fig. 4:**
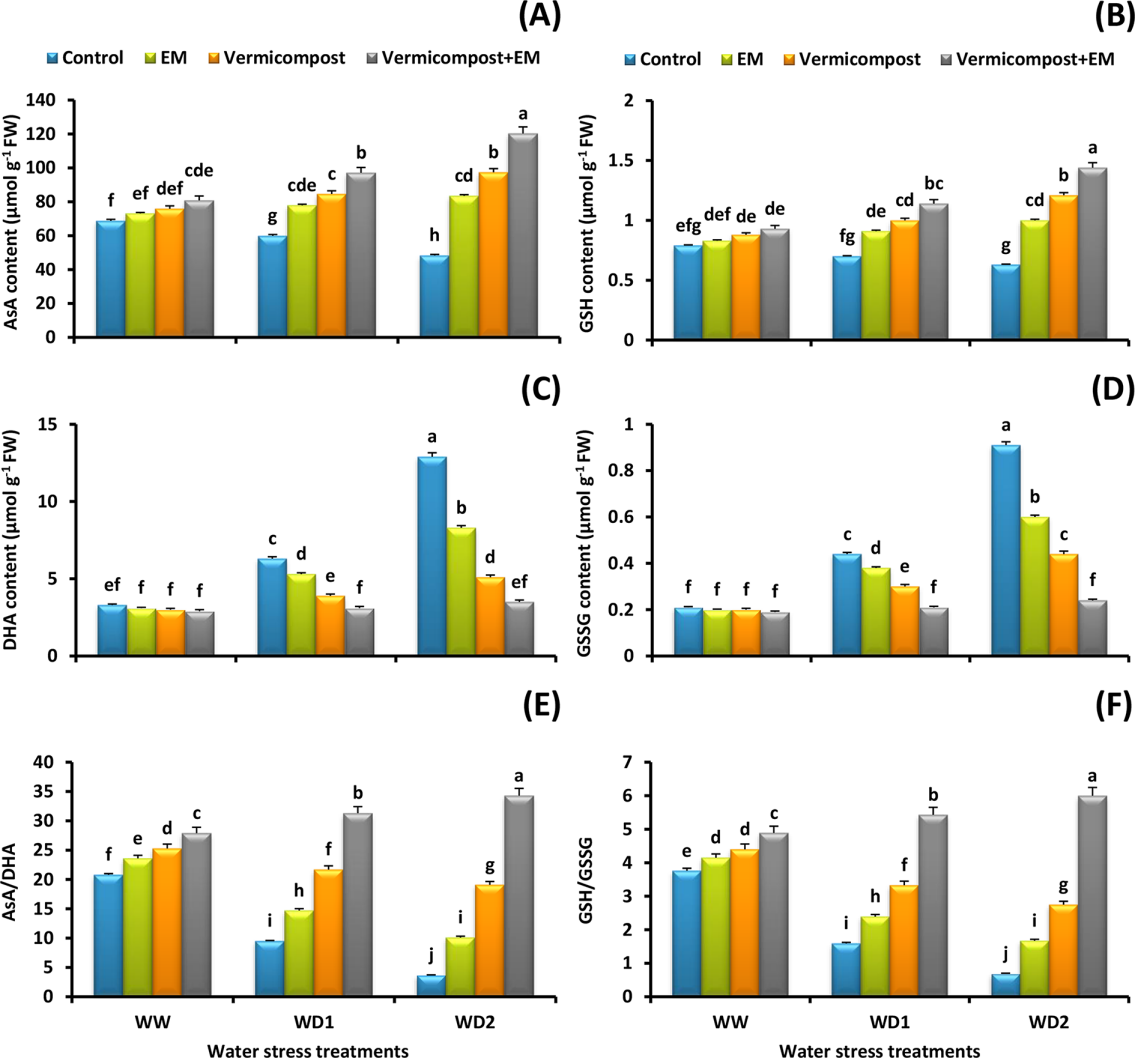
Effect of effective microorganisms (EM), vermicompost, and their interaction on the: **A** ascorbate (AsA), **B** reduced glutathione (GSH), **C** dehydroascorbate (DHA), and **D** oxidized glutathione (GSSG) contents, as well as **E** AsA/DHA and **F** GSH/GSSG ratios in leaves of wheat plants grown under different water stress conditions [well-watered (WW), water deficit stress (WD1; 70% of field capacity), and water deficit stress (WD2; 30% of field capacity)]. Vertical bars represent ± standard error of the mean (*n* = 4). Different letters indicate significant differences between treatments at *p* < 0.05 level according to LSD test

To elucidate the mechanism underlying how vermicompost and/or EM treatments modulate antioxidant capacity, the content of different antioxidant molecules in leaves of wheat plants were assayed. Our findings showed that the content of AsA and GSH was significantly declined by 30 and 20%, respectively under a severe (30% FC) water deficit condition relative to the non-stressed plants. However, vermicompost and/or EM applications significantly mitigated the deleterious impact of water stress and assisted in regenerating their content (Fig. 4A and B). The greatest contents were obtained by vermicompost and EM combined treatment. It significantly improved the content of AsA by 18, 62, and 149% and GSH by 18, 63, and 129% compared to the untreated plants under well-watered and water-stressed (70% and 30% FC) conditions, respectively. Moreover, a significant increase in the content of DHA (291%) and GSSG (333%) was observed under a severe (30% FC) water deficit condition relative to the non-stressed plants. On the contrary, vermicompost and/or EM treatments significantly decreased their levels (Fig. 4C and D). Interestingly, the ratio of AsA/DHA and GSH/GSSG was significantly increased by vermicompost and/or EM applications under both non-stressed and stressed conditions (Fig. 4E and F). In comparison to the untreated plants, combined treatment under the severe (30% FC) water stress condition significantly (*p* < 0.05) increased the AsA/DHA ratio (827%) and GSH/GSSG ratio (770%) in wheat leaves.

### Total phenols and flavonoids content

**Fig. 5:**
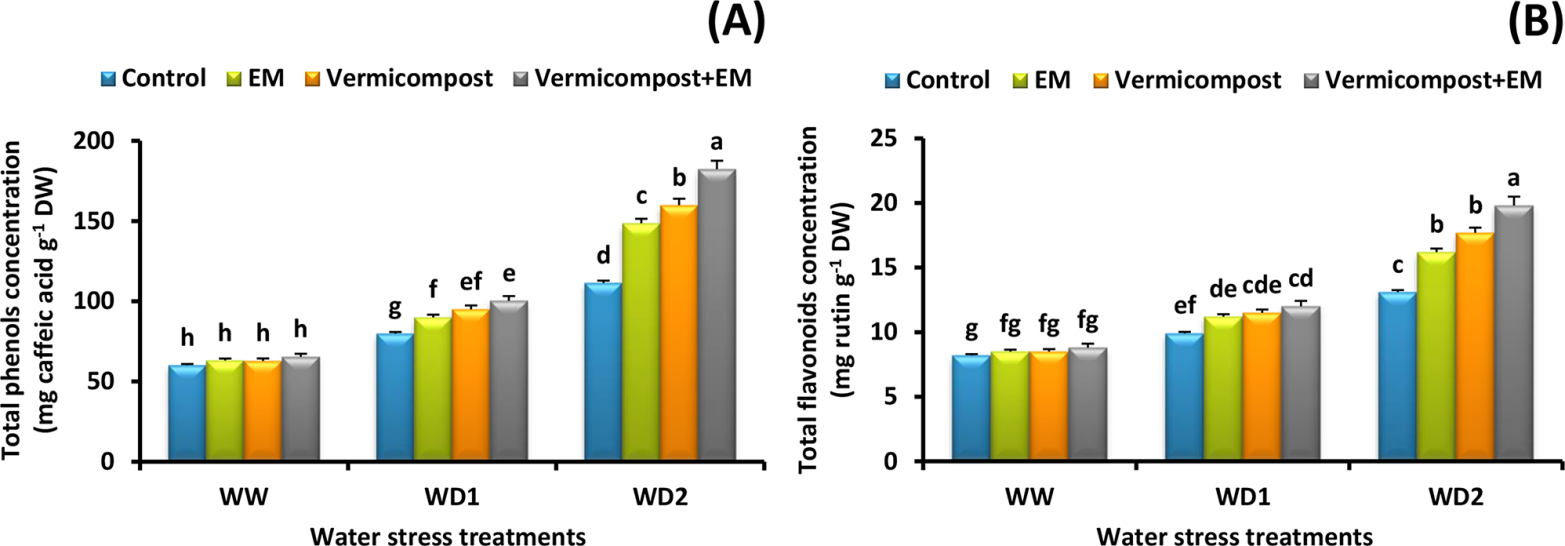
Effect of effective microorganisms (EM), vermicompost, and their interaction on the: **A** total phenols and **B** total flavonoids concentration in leaves of wheat plants grown under different water stress conditions [well-watered (WW), water deficit stress (WD1; 70% of field capacity), and water deficit stress (WD2; 30% of field capacity)]. Vertical bars represent ± standard error of the mean (*n* = 4). Different letters indicate significant differences between treatments at *p* < 0.05 level according to LSD test

As shown in Fig. 5A and B, drought conditions significantly increased both total phenols and flavonoids content in wheat leaves. A severe (30% FC) water deficit condition caused 85 and 60% increase in their concentrations, respectively relative to the non-stressed plants. Vermicompost and/or EM applications further increased their accumulation in water-stressed plants. Wheat plants subjected to a severe (30% FC) water deficit condition and received a combined treatment accumulated much more phenols and flavonoids than other treated plants. The combined treatment significantly enhanced phenolic and flavonoid concentrations by 64 and 51% respectively, in plants grown under a severe (30% FC) water deficit condition, compared with control plants.

### Methylglyoxal content and glyoxalase activity

**Fig. 6:**
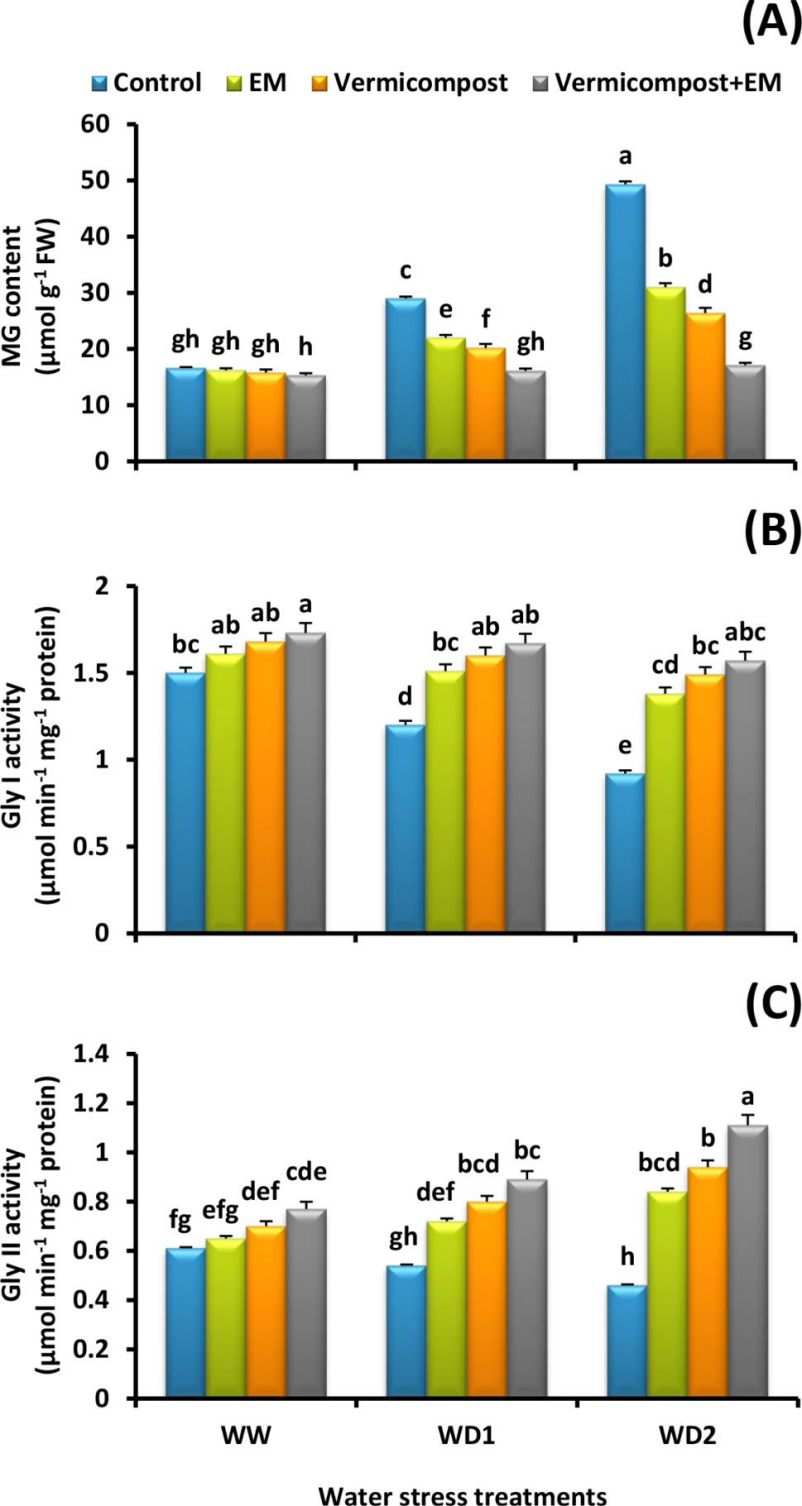
Effect of effective microorganisms (EM), vermicompost, and their interaction on the: **A** methylglyoxal (MG) content, as well as **B** glyoxalase I (Gly I) and **C** glyoxalase II (Gly II) activities in leaves of wheat plants grown under different water stress conditions [well-watered (WW), water deficit stress (WD1; 70% of field capacity), and water deficit stress (WD2; 30% of field capacity)]. Vertical bars represent ± standard error of the mean (*n* = 4). Different letters indicate significant differences between treatments at *p* < 0.05 level according to LSD test

In view of the effect of water deficit environments on glyoxalase machinery, the results summarized in Fig. 6A show that a severe (30% FC) water deficit condition significantly increased MG content by 197% compared to control plants. However, vermicompost and/or EM applications significantly decreased its content under water scarcity. The maximum ameliorative effect was detected by vermicompost and EM co-application. It significantly decreased the MG content by 8, 45, and 65% compared to the untreated plants under well-watered and water-stressed (70% and 30% FC) conditions, respectively. Interestingly, the glyoxalase system, comprising the Gly I and Gly II enzymes, effectively eliminates cytotoxic MG. The activity of both Gly I and Gly II were decreased by 39 and 25%, respectively under a severe (30% FC) water deficit condition compared with control plants. However, vermicompost and/or EM applications significantly increased their activities (Fig. 6B and C). The highest activities were achieved by vermicompost and EM combined treatment. In comparison to the untreated plants, combined treatment under the severe (30% FC) water stress condition significantly (*p* < 0.05) increased the activity of Gly I (71%) and Gly II (141%) in wheat leaves.

### Osmotic regulatory substances content

**Fig. 7:**
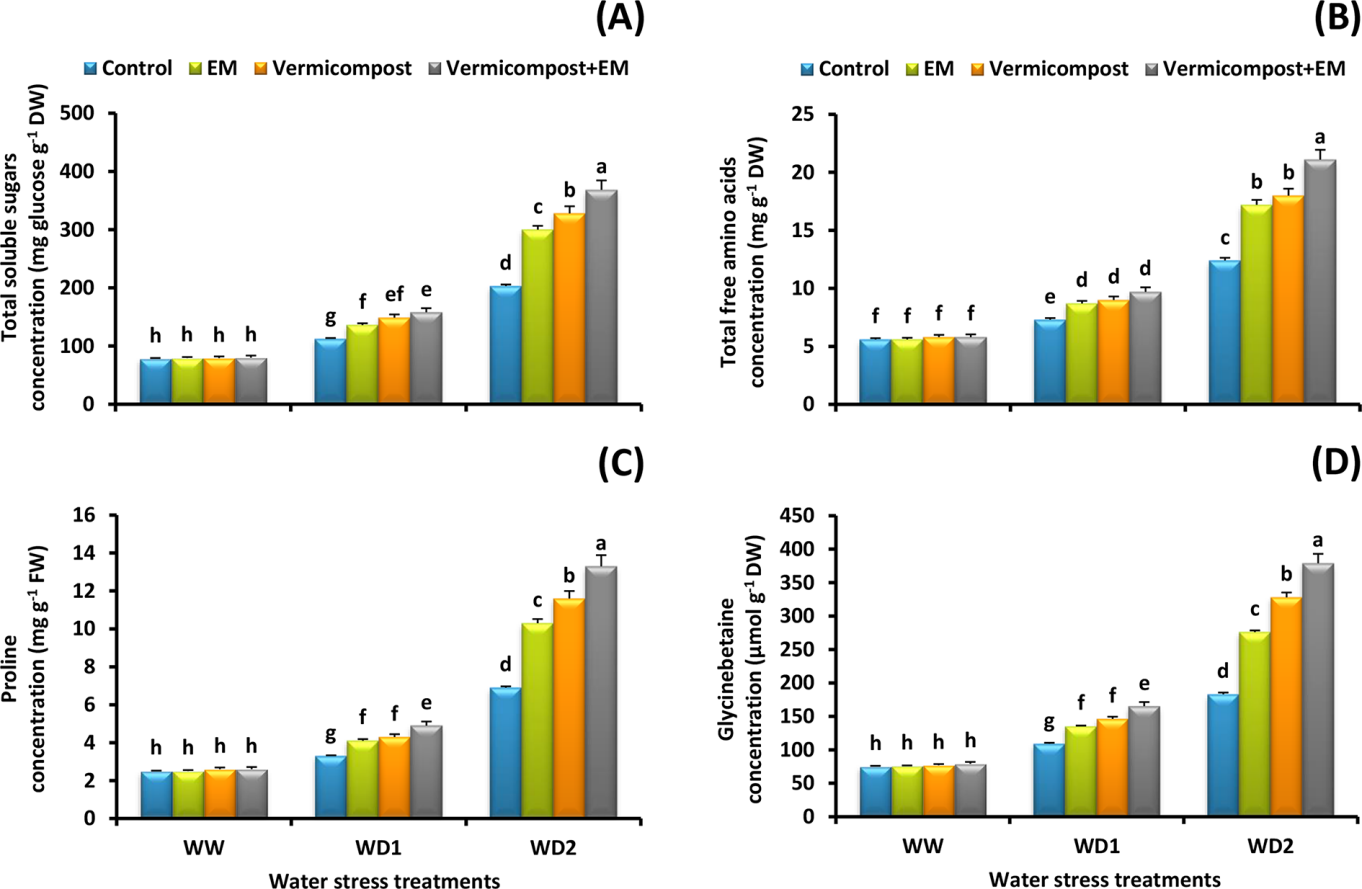
Effect of effective microorganisms (EM), vermicompost, and their interaction on the: **A** total soluble sugars, **B** total free amino acids, **C** proline, and **D** glycinebetaine concentrations in leaves of wheat plants grown under different water stress conditions [well-watered (WW), water deficit stress (WD1; 70% of field capacity), and water deficit stress (WD2; 30% of field capacity)]. Vertical bars represent ± standard error of the mean (*n* = 4). Different letters indicate significant differences between treatments at *p* < 0.05 level according to LSD test

In view of the effect of water deficiency on leaf organic solutes accumulation, it was postulated that an increase of 159, 121, 176, and 144% was recorded in the amount of leaf total soluble sugars, free amino acids, proline, and glycinebetaine, respectively in plants subjected to a severe (30% FC) water deficit condition relative to the non-stressed plants. Nevertheless, this increase was further amplified upon supplementation of vermicompost and/or EM under water deficit conditions (Fig. 7A-D). The maximum values were detected by vermicompost and EM co-application. In comparison to the untreated plants, combined treatment under the severe (30% FC) water stress condition significantly (*p* < 0.05) increased the concentration of soluble sugars (81%), free amino acids (70%), proline (93%), and glycinebetaine (107%) in wheat leaves.

### Relative water content

**Fig. 8:**
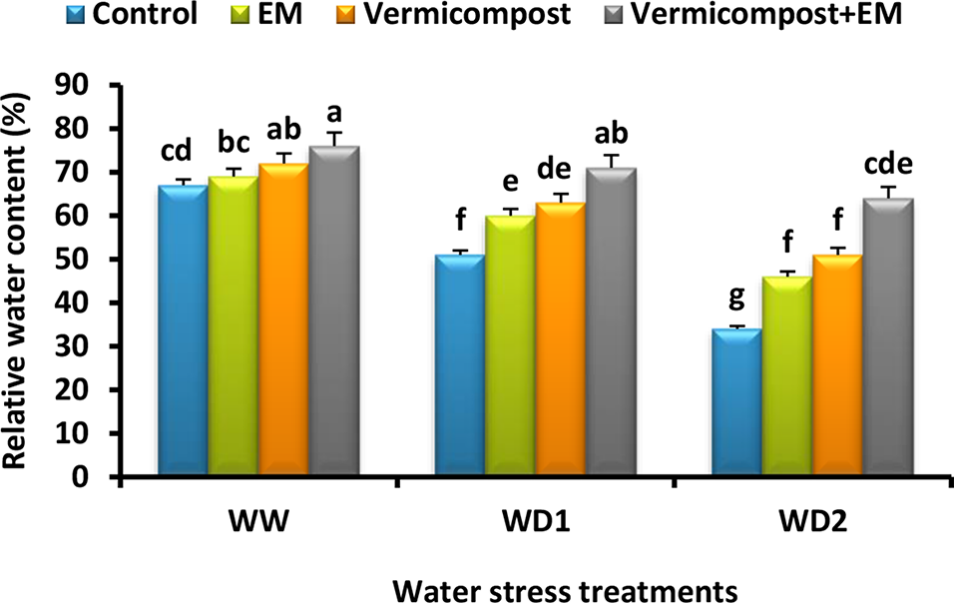
Effect of effective microorganisms (EM), vermicompost, and their interaction on the relative water content (%) in leaves of wheat plants grown under different water stress conditions [well-watered (WW), water deficit stress (WD1; 70% of field capacity), and water deficit stress (WD2; 30% of field capacity)]. Vertical bars represent ± standard error of the mean (*n* = 4). Different letters indicate significant differences between treatments at *p* < 0.05 level according to LSD test

In our study, Fig. 8 reveals that water scarcity significantly decreased the leaf RWC of wheat plants compared to control ones. Plants grown under a severe (30% FC) water deficit condition had significantly lower RWC (49%) than non-stressed plants. On the contrary, vermicompost and/or EM applications significantly increased RWC under both non-stressed and stressed conditions. The maximum value was detected by vermicompost and EM co-application. It significantly enhanced the RWC by 13, 39, and 88% compared to the untreated plants under well-watered and water-stressed (70% and 30% FC) conditions, respectively.

## Discussion

Drought is one of the most serious environmental stressors that harms environmentally sustainable systems and food security [4, 9]. To reduce the impact of environmental stresses, sustainable agriculture systems must employ environmental-friendly amendments [56]. Vermicompost and EM are excellent plant growth promoters because they have the intrinsic potential to boost photosynthetic capacity, nutrient absorption, water use efficiency, cell membrane stability, and free radical detoxification under water stress circumstances [21, 25, 27, 31, 32]. Nonetheless, no data on the effect of employing vermicompost in conjunction with EM on wheat drought tolerance are currently available. The current work demonstrates that using vermicompost in combination with EM can activate antioxidant defense and glyoxalase systems, as well as buildup osmotic regulatory substances, resulting in less oxidative stress damage caused by water stress in wheat plants. This research gives novel insights into the mechanisms of water stress relief in wheat using vermicompost and EM treatments.

In the following experiment, drought hampered wheat growth and productivity. This result is consistent with the previous findings of [8, 10, 21]. This decline may be explained by a decrease in tissue water content, which reduces cell turgor pressure and inhibits cell enlargement and expansion [57], a decline in photosynthetic activity, and biomass production [5, 15], a decrease in nutrient metabolism and ion uptake [22, 58], and an increase in oxidative damage due to disruption of the antioxidant defense system [4, 9]. Interestingly, we observed that applying vermicompost and/or EM to water-stressed plants improved plant growth and production attributes (Fig. 1A-F). Previous studies indicated that vermicompost is regarded as an excellent nutrient-rich organic fertilizer and soil conditioner that can stimulate plant growth and production under water stress conditions [8, 26, 59–61] by improving soil organic carbon storage and hence its porosity, aeration, and drainage [62, 63], soil water holding capacity [20, 21], soil microbial activity [18, 64], nutrients availability such as nitrates, exchangeable phosphate, soluble potassium, calcium, and magnesium, as well as growth hormones and beneficial enzymes [18, 25], antioxidant system capacity for effective ROS scavenging [22, 23], and photosynthetic activity [21, 65]. Additionally, EM application can increase crop yield during water scarcity by improving microbial activity and soil fertility [32, 66], soil water holding capacity [28, 31], and plant growth-promoting substances (amino acids, vitamins, sugars, lactic acids, enzymes, hormones, …etc.) production [30, 33, 35]. Furthermore, EM can benefit stressed plants by supplying nutrients, enhancing nutrient absorption, improving photosynthesis, boosting antioxidant enzyme activity, and inducing phytohormonal production [26, 29–31, 33, 34]. Therefore, it is worth noting that vermicompost and EM appear to be promising alternatives for developing specialized eco-friendly fertilizer to improve wheat drought tolerance.

Water stress is one of the most widespread environmental problems we face today affecting not only plant development and agricultural productivity, but also causing oxidative damage to plant tissues [4]. Drought stress causes an imbalance between ROS generation and scavenging [5]. In this work, we found that drought-stressed wheat plants accumulate more O_2_^• −^ and H_2_O_2_ than the unstressed plants. These findings are in accordance with [10, 11] who detected a greater ROS accumulation in plants exposed to water-scare environment. The decrease in antioxidant enzymes’ ability to scavenge ROS may be the cause of this accumulation in wheat leaves. This circumstance might accelerate oxidative stress, resulting in poor growth performance. In contrast, the current research also demonstrated that the application of vermicompost and/or EM greatly reduces the generation of ROS molecules in stressed plants (Fig. 2A and B), which could be attributed to an increase in antioxidant enzymes (SOD, CAT, POD, APX, GPX, MDHAR, DHAR, and GR) activity. Our findings concur with those of [23, 28, 29, 35], who found that applying vermicompost or EM improves plant antioxidant activity under stressful conditions.

Our findings also revealed an increase in MDA content in wheat leaves under water scarcity, which can be explained by an increase in oxidative cellular damage and a decrease in ROS scavenging activity. The results we obtained align with the observations made by [4, 10, 15], who noted elevated MDA content in water-stressed plants. In contrast, stressed plants treated with vermicompost and/or EM showed lower levels of MDA than stressed untreated ones (Fig. 2C). The increased activity of antioxidant enzymes in these plants may be the cause of this decline. Although water stress caused ROS formation, the increased antioxidant enzyme activity in stressed-treated plants cleaned the ROS, preventing membrane lipid peroxidation damage in the leaves. Previous studies have reported comparable results, suggesting that the application of vermicompost can safeguard the integrity of cell membranes by reducing ROS and MDA contents [23, 65]. Likewise, prior reports on the effects of EM on stressed plants found a decrease in MDA and H_2_O_2_ contents [30]. Actually, [67] proposed that improving membrane repair and triggering antioxidant enzyme response helps to decrease MDA content, providing protection against oxidative damage.

In response to drought stress and ROS accumulation, plants have evolved a variety of enzymatic and non-enzymatic antioxidant systems [11]. In the following experiment, drought stress significantly reduced the activity of SOD, CAT, and POD, resulting in overproduction of ROS. These results are in accordance with [4, 5, 10, 13]. Interestingly, we found that applying vermicompost and/or EM treatments increases their activities (Fig. 3A-C), suggesting a reduction in O_2_^• −^ and H_2_O_2_ buildup. Actually, the initial step in reducing ROS stress is activating SOD, which converts O_2_^• −^ to H_2_O_2_ and that can be converted to water and oxygen by CAT, POD, APX, and GR [68–70]. Prior studies have shown that vermicompost plays a crucial role in enhancing plant drought tolerance by altering ROS level [22, 23]. In this regard, [71] previously observed that vermicompost comprises micronutrients such as iron that act as a prosthetic group of hemoproteins like SOD, CAT, and POD. Additionally, [28, 29, 33, 35] revealed that EM application enhanced antioxidant enzyme activity in stressed plants. It is noteworthy that greater antioxidant enzyme activity in drought-stressed vermicompost- and/or EM-treated plants may be associated to plant drought tolerance, which can detoxify ROS and reduce membrane lipid peroxidation.

The AsA-GSH pathway actively participates in ROS detoxification and water stress tolerance [4, 11]. In our experiment, all the major AsA-GSH pathway enzymes; APX, GPX, MDHAR, DHAR, and GR, were significantly downregulated in response to drought stress; however, applying vermicompost and/or EM treatments strongly upregulated their activity (Fig. 3D-H). These results are in accordance with [23, 28, 72] who found that the AsA-GSH cycle capacity is increased by vermicompost or EM treatment. Additionally, we also observed a sharp decline in the content of GSH and AsA in water-stressed plants. In fact, during drought stress, a plant’s capacity to produce antioxidants is hampered by the generation of ROS, which disturbs redox homeostasis [5, 11]. Interestingly, vermicompost and/or EM played a crucial role in reducing the harm caused by ROS by inducing GSH and AsA pools and keeping their pools under water-scare environment (Fig. 4A and B). The findings obtained by [28, 29, 33], are consistent with our results. The current study’s results thus support the idea that plants treated with vermicompost and/or EM have a greater potential to control the ratio of ROS production to ROS elimination by enhancing the enzymatic and non-enzymatic antioxidant capacity. It is worth noting that higher enzymatic antioxidant activity and non-enzymatic antioxidant content as a result of vermicompost and/or EM supplementations are associated with reduced lipid peroxidation, which helps in cell membrane stabilization and function maintenance, thereby improving plant growth performance and productivity.

Our findings further indicate that the concentration of phenolic compounds and flavonoids rose under water deficiency, and that this rise was amplified by the addition of vermicompost and/or EM (Fig. 5A and B). Our findings are consistent with those of [8, 28, 33, 73] who found that the production of phenolic compounds and flavonoids was favorably linked with vermicompost or EM treatments under stressful conditions. Indeed, there is a clear link between stress tolerance and phenolic and flavonoid compounds, which can play an important role in ROS detoxification, lipid peroxidation inhibition, and cell membrane stabilization [74, 75]. As a result, it is worth noting that boosting phenolic and flavonoid compounds can help vermicompost- and EM-treated plants to achieve drought tolerance.

Drought stress also disturbed the glyoxalase system, resulting in increased MG synthesis; however, overexpression of Gly I and Gly II can reduce MG overaccumulation [4]. In our investigation, drought stress enhanced MG production while decreasing Gly I and Gly II activity. However, vermicompost and/or EM treatments improve the plant’s ability to withstand drought stress by reducing MG production via upregulating Gly I and Gly II activities (Fig. 6A-C). According to research by [76], GSH played a central role in coordinating antioxidant defense and glyoxalase systems through its interaction with ROS and MG detoxification, which was vital for drought-induced oxidative stress tolerance.

The current study’s findings demonstrated that drought-stressed plants’ leaves had an accumulation of osmolytes, such as total soluble sugars, free amino acids, glycinebetaine, and proline. This result is in line with previous studies that shown elevated proline, glycinebetaine, and soluble sugars accumulation under stressful conditions, indicating their roles as protective agents [4, 15, 77, 78]. Additionally, a number of studies have discovered a rise in the concentration of free amino acids during water scarcity, which could be attributed to an increase in the activity of enzymes that degrade protein and a decrease in the synthesis of new proteins [79, 80]. Interestingly, under water stress, the study’s findings indicated a greater improvement in the acquisition of osmotic regulatory substances in plants treated with vermicompost and/or EM (Fig. 7A-D). Vermicompost or EM applications have been shown in prior research to increase the accumulation of compatible solutes in stressed plants, which can be crucial in regulating the water relationship, preserving the integrity and stability of the cell membrane, and preventing the oxidative damage induced by ROS [21, 22, 28]. Therefore, it is important to note that plants treated with vermicompost and/or EM showed improved tolerance to drought stress due to increased osmolyte accumulation, which helped to maintain a better plant water status. This, in turn, led to comparatively less damage from oxidative stress and increased grain yield.

In our investigation, wheat leaves exposed to drought stress showed a considerable drop in RWC when compared to control ones. This decline appears to be related to impaired plasma membrane permeability, which causes excessive water loss. As water deficiency increased, reduced leaf RWC was also noted by [4, 15, 22]. However, the study’s findings showed that applying vermicompost and/or EM improved the wheat’s capacity to retain RWC (Fig. 8), hence reducing the detrimental impacts of drought stress. Our results align with earlier studies that have observed an improvement in RWC by vermicompost or EM supplementations under stressful conditions [21, 22, 28, 29]. Vermicompost has been shown in previous studies to increase the amount of water that reaches roots because of its high water-holding capacity and the presence of mycorrhizal fungi and other microorganisms [81]. Adding vermicompost [19–21] or EM [27, 30] has also been shown in earlier studies to enhance soil moisture characteristics by optimizing soil structure, which eventually results in higher leaf RWC. Actually, the use of these beneficial amendments in the current study demonstrated practical methods for lessening the detrimental effects of drought stress by raising tissue water content. This may be because of their effects on osmolyte and antioxidant compound accumulation, which can modulate osmotic pressure and mitigate oxidative damage, protecting cellular turgor pressure and membrane integrity against damage from water stress.

**Fig. 9:**
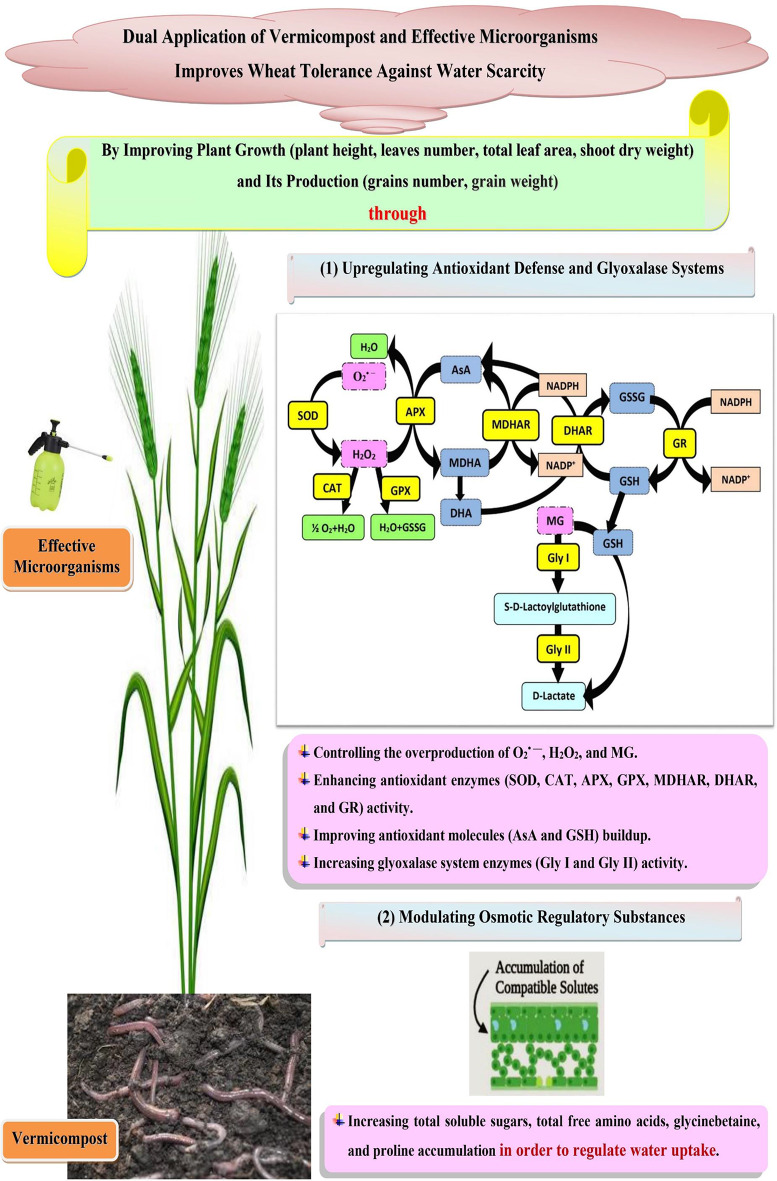
Co-application of vermicompost and effective microorganisms improves wheat tolerance against water scarcity by upregulating antioxidant defense and glyoxalase systems and modulating osmotic regulatory substances

In sum, our findings emphasize the critical function that vermicompost and/or EM treatments play in enhancing wheat’s tolerance to drought by promoting plant growth and productivity through the regulation of the antioxidant defense and glyoxalase systems, as well as the improvement of osmolyte accumulation (Fig. 9). The innovative use of vermicompost and EM dual treatment has significant potential to preserve wheat crop from oxidative damage and pave the path for sustainable agricultural progress.

## Conclusion

The current study aimed to alleviate the negative impacts of water stress and enhance wheat productivity by vermicompost and EM. Wheat plants exposed to drought experienced oxidative damage, as evidenced by increases in superoxide radical, hydrogen peroxide, methylglyoxal, and lipid peroxidation, as well as decreases in antioxidant and glyoxalase system enzyme activity, antioxidant molecule content, and relative water content. Alternatively, the co-application of vermicompost and EM improved wheat drought tolerance by increasing the activity of enzymes linked to drought tolerance, the amount of antioxidant molecules, and the acquisition of osmotic regulatory substances. Moreover, the glyoxalase system improved significantly with the dual application of vermicompost and EM by reducing the overproduction of methylglyoxal and raising the activity of the system’s enzymes, which strengthened wheat’s tolerance to oxidative damage. Taken together, these results provide novel insights and improve our understanding of the mechanisms underlying vermicompost-EM synergetic relationship to enhance plant drought tolerance. The vermicompost and EM combined treatment can then offer a financially and environmentally responsible way to promote plant growth and enhance the tolerance to drought stress in a sustainable manner. The results of this study are a step toward sustainable agriculture and may help protect plants from drought stress.

## Electronic supplementary material

Below is the link to the electronic supplementary material.

Supplementary Material 1

## Data Availability

All data generated or analyzed during this study are included in this published article.

## Abbreviations

EM: Effective Microorganisms
ROS: Reactive Oxygen Species
WW: Well-Watered; 100% of Field Capacity
WD1: Water Deficit Stress; 70% of Field Capacity
WD2: Water Deficit Stress; 30% of Field Capacity
O_2_^• −^: Superoxide Radical
H_2_O_2_: Hydrogen Peroxide
MDA: Malondialdehyde
SOD: Superoxide Dismutase
CAT: Catalase
POD: Peroxidase
APX: Ascorbate Peroxidase
MDHAR: Monodehydroascorbate Reductase
DHAR: Dehydroascorbate Reductase
GR: Glutathione Reductase
GPX: Glutathione Peroxidase
AsA: Ascorbate
DHA: Dehydroascorbate
GSH: Glutathione
GSSG: Oxidized Glutathione
MG: Methylglyoxal
Gly I: Glyoxalase I
Gly II: Glyoxalase II
RWC: Relative Water Content
