# Supplementary material for: An innovative, sustainable, and environmentally friendly approach for wheat drought tolerance using vermicompost and effective microorganisms: upregulating the antioxidant defense machinery, glyoxalase system, and osmotic regulatory substances
Source: BMC Plant Biol. 2024 Sep 17;24:866. doi: 10.1186/s12870-024-05550-2 (PMC11406802; doi:10.1186/s12870-024-05550-2)
Supplement: Supplementary file 1 — Supplementary Material 1 [file 12870_2024_5550_MOESM1_ESM.docx]

**Table S.** *P*-values from a two-way analysis of the assessed parameters in wheat plants as influenced by different applications at various drought treatments. *P*-values in bold are considered statistically significant (<0.05, n = 4). 'D' denotes drought treatments effect; 'T' denotes different applications effect; D x T denotes variables’ interaction effect

|  | **Main-factor effects** | | **Significant interaction** |
| --- | --- | --- | --- |
|  | **D** | **T** | **D x T** |
| **Plant height** | <0.0001 | <0.0001 | - |
| **Leaves number** | <0.0001 | <0.0001 | - |
| **Total leaf area** | <0.0001 | <0.0001 | 0.1138 |
| **Shoot dry weight** | <0.0001 | <0.0001 | - |
| **Grains number** | <0.0001 | <0.0001 | - |
| **Grain yield** | <0.0001 | <0.0001 | - |
| **Superoxide content** | <0.0001 | <0.0001 | 0.0021 |
| **Hydrogen peroxide content** | <0.0001 | <0.0001 | <0.0001 |
| **Lipid peroxidation** | <0.0001 | <0.0001 | <0.0001 |
| **Superoxide dismutase activity** | 0.0034 | <0.0001 | <0.0001 |
| **Catalase activity** | <0.0001 | <0.0001 | <0.0001 |
| **Peroxidase activity** | <0.0001 | <0.0001 | <0.0001 |
| **Ascorbate peroxidase activity** | 0.0002 | <0.0001 | <0.0001 |
| **Glutathione peroxidase activity** | 0.1058 | <0.0001 | <0.0001 |
| **Monodehydroascorbate reductase activity** | <0.0001 | <0.0001 | <0.0001 |
| **Dehydroascorbate reductase activity** | <0.0001 | <0.0001 | <0.0001 |
| **Glutathione reductase activity** | 0.0122 | <0.0001 | <0.0001 |
| **Ascorbate content** | <0.0001 | <0.0001 | <0.0001 |
| **Dehydroascorbate content** | <0.0001 | <0.0001 | <0.0001 |
| **AsA/DHA ratio** | <0.0001 | <0.0001 | <0.0001 |
| **Reduced glutathione content** | 0.0002 | <0.0001 | 0.0010 |
| **Oxidized glutathione content** | <0.0001 | <0.0001 | <0.0001 |
| **GSH/GSSG ratio** | <0.0001 | <0.0001 | <0.0001 |
| **Total phenols concentration** | <0.0001 | <0.0001 | <0.0001 |
| **Total flavonoids concentration** | <0.0001 | <0.0001 | 0.0012 |
| **Methylglyoxal content** | <0.0001 | <0.0001 | <0.0001 |
| **Glyoxalase I activity** | <0.0001 | <0.0001 | 0.0756 |
| **Glyoxalase II activity** | 0.0004 | <0.0001 | 0.0015 |
| **Total soluble sugars concentration** | <0.0001 | <0.0001 | <0.0001 |
| **Total free amino acids concentration** | <0.0001 | <0.0001 | <0.0001 |
| **Proline concentration** | <0.0001 | <0.0001 | <0.0001 |
| **Glycinebetaine concentration** | <0.0001 | <0.0001 | <0.0001 |
| **Relative water content (%)** | <0.0001 | <0.0001 | <0.0001 |
